# Experimental Analysis and Simulation of Novel Technical Textile Reinforced Composite of Banana Fibre

**DOI:** 10.3390/ma12071134

**Published:** 2019-04-07

**Authors:** Mario D. Monzón, Rubén Paz, Martí Verdaguer, Luis Suárez, Pere Badalló, Zaida Ortega, Noelia Diaz

**Affiliations:** 1 Mechanical Engineering Department, Universidad de Las Palmas de Gran Canaria, Edificio de ingenierías, Campus de Tafira Baja, 35017 Las Palmas, Spain; ruben.paz@ulpgc.es (R.P.); luis.suarez@ulpgc.es (L.S.); 2 Leitat Technological Centre, C de la Innovació, 2.08225 Terrassa, Spain; martivema@gmail.com (M.V.); pbadallo@leitat.org (P.B.); 3 Processes Engineering Department, Universidad de Las Palmas de Gran Canaria, Edificio de ingenierías, Campus de Tafira Baja, 35017 Las Palmas, Spain; zaida.ortega@ulpgc.es (Z.O.); noelia.diaz@ulpgc.es (N.D.)

**Keywords:** natural fibre composite, banana fibre, computational modelling, mechanical testing, compression moulding

## Abstract

The use of natural fibres allows reducing environmental impact, due to their natural renewable origin and the lower energy needed for their production and processing. This work presents the mechanical characterization of a newly developed technical textile, with banana fibre treated by enzymes, comparing experimental results with numerical simulation based on the definition of the unit cell at micromechanical level. The experimental test shows that the composite with the fabric of banana fibre presents worse mechanical behaviour than the one with commercial flax fibre. The presence of wool, necessary for producing the yarn, reduces the mechanical properties of the banana textile. The numerical simulation had an acceptable error compared with the experimental results, with a global average error of 9%, showing that the predictive modelling based on the multiscale method is suitable for the design process of this kind of composite.

## 1. Introduction

The use of natural fibres allows the reduction of environmental impact, due to their natural renewable origin and the lower energy needed for their production and processing (4 MJ/kg of natural fibre versus 30 MJ/kg of glass fibre or 130 MJ/kg of carbon fibre) [1]. Natural fibres have been traditionally used for the production of ropes or sacs, but in recent years it has started to be used for technical applications, both for composite materials and for the production of speciality papers. Banana fibre is a cellulosic fibre, obtained from the leaves of the pseudostem of the plant. Banana tree is a monocot plant, from the genus *Musa*, in the Musaceae family. These plants have no wood or a real trunk; the pseudostem is made of different leaves tight together. Once the fruits have been harvested, the plant produces one or various shoots, which will replace the original plant, dead after the fruits production. Pseudostems are usually left in the plantation, producing a problem for the forthcoming harvesting. Banana fibre has 60%–65% cellulose, 6%–8% hemicellulose, and 5%–10% lignin [2]. The “Integrated and Advanced Manufacturing” research group of the University of Las Palmas de Gran Canaria has been researching in the process of extracting the banana fibre from the species grown in the Canary Islands, with application to several materials such as reinforcement for composite materials and cellulose paste. The developed technology for extracting the fibre has been patented at international level [3]. Among the different applications of banana fibre (as reinforcement of composite material), to the production of continuous yarn and technical textile is considered the most promising [4].

One relevant issue, for all the polymeric composites in general and natural fibre composites in particular, is the efficiency of the adhesion fibre-matrix. Many works have been carried out to study different fibre treatments to improve this integration. These treatments can be physical, chemical, or biological, being the chemical ones the most commonly studied and applied, such as alkali with NaOH, among others. In a review by Kalia about treatments in natural fibre composites [5], most of the research showed that the chemical treatments decreased the strength properties because of the breakage of the bond structure and also because of the disintegration of the non-cellulosic materials. In this line, Eddine et al. [6] reported that alkali pre-treatments decrease the properties of elementary fibres by damaging polysaccharides in surface. However, the adhesion fibre-matrix is improved, and thus the quality of the composite. Regarding the enzymatic treatment of natural fibres, which is very common in the textile industry, some studies show pectinase and xylanase as the most suitable enzymes for fibre extraction [4]. In the present work, the banana fibre has been treated by enzymes to produce the yarns.

Additionally, several works have been carried out to develop and test fabrics of natural fibre integrated into a polymeric matrix. For instance, Madsen et al. [7] detailed the characterization and analyses of the properties and performance of unidirectional flax yarn/thermoplastic polyester composites. By applying the model of mixtures, they determined the anisotropic effect of the composite due to the unidirectional positioning of the fibre, concluding that the ratio of anisotropy between two directions was about 13 (different *E* modulus). Muralidhar [8] determined the tensile and compressive properties of flax-plain weave preform reinforced epoxy composite. Depending on the laminate, this author measured different levels of anisotropy, mainly for compression testing, where both the *E* modulus and the maximum strength were quite anisotropic. Not only the mechanical characterization has been studied in fabric natural fibre composites, but also some relevant studies, for marine applications, have been done, testing the effects of water immersion ageing on the mechanical properties of flax and jute fibre [9]. These authors evaluated the behaviour of woven fabric flax and jute fibre reinforced bioresin-based epoxy biocomposite, after water immersion, by nanoindentation and flexural testing. However, no studies related to the development of fabrics of banana fibre by the method proposed in the present research have been reported.

Different predictive models for textile composites have been developed over the years, starting from analytical models [10] and later improved through the use of finite element analysis (FEA) [11]. The increasing capabilities of numerical simulations have led to the evolution of new approaches based on FEA for the simulation of 2D braided composites, as reviewed in [12]. Other authors have also implemented user-defined sub-routines in standard FEA software, such as Abaqus, to simulate the mechanical behaviour of carbon or glass fibre reinforced polymer (CFRP and GFRP respectively) [13]. Through user-defined subroutines, these authors established the orientation and anisotropic properties of the material composite. However, the current trend is the use of the homogenization approach [14] through the simulation of unit cells. This consists in analysing the behaviour of a representative unit cell of the composite material. Afterwards, the obtained anisotropic properties are applied to the entire composite body. This approach allows the determination of the mechanical properties of a heterogeneous material through the upscaling of a unit cell. The advantage of this method is that the unit cell properties can be finely determined and then extrapolated, while the simulation of a complete composite material with the accuracy of the cell unit definition is unfeasible. According to the literature, WiseTex [15,16] and Multiscale Designer [17] are the most common software used for this purpose.

This paper presents a part of the work carried out to study the viability of using novel banana fabric as reinforcement of a polymeric matrix processed under compression moulding and the comparison with predictive modelling based on the multiscale method (Multiscale Designer^TM^, Altair, Troy, MI, USA)

## 2. Materials and Methods

### 2.1. Technical Textile

In the context of this project, the research groups of University of Las Palmas de Gran Canaria and Leitat developed a novel technical textile based on banana fibre extracted from pseudostems by a patented process [3] and treated by enzymatic process. The enzymatic process, with poligalacturonase for 6 h at 45 °C and pH = 4.5, allowed the production of continuous yarn of banana fibre mixed with 50% of wool [4]. In order to improve the stability of the yarn, the banana fibre yarn was twisted with multifilament of polypropylene. The linear mass density of the yarn (TEX, mass in grams per 1000 m) was 157, with an average diameter of the banana yarn of 0.24 mm and the one twisted with multifilament of PP 0.36 mm.

Tensile tests of 16 samples of banana yarns were performed to study the mechanical behaviour under tensile force. The test was carried out under the standard UNE-EN ISO 2062-2010 [18], method A, using an Instron constant elongation gradient dynamometer (VCA, Norwood, MA, USA), class 0.5. The resulting data were treated considering the yarn as a single continuous material. Samples were stored for 24 h, at 20 ± 2 °C, 65% ± 4% relative humidity (EN ISO 139 [19]). The length of samples was 250 mm and speed of testing 250 mm/min. The yield strength was determined by finding the stress in which the difference of strain between the curve and the line drawn by the Young’s modulus exceeds 3%. The average results were 669 MPa for Young modulus and 36.1 MPa for Yield strength.

**Figure 1:**
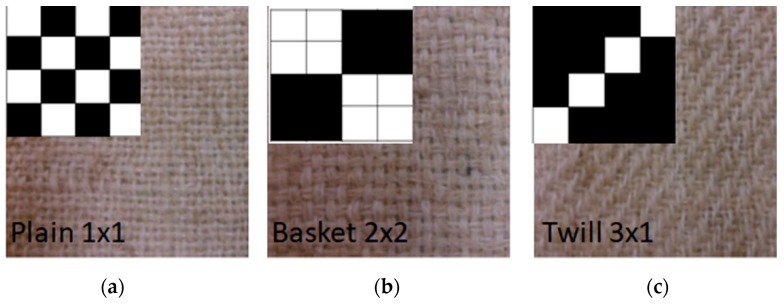
Configurations of technical textile of banana. (**a**) Plain; (**b**) Basket and (**c**) Twill.

**Figure 2:**
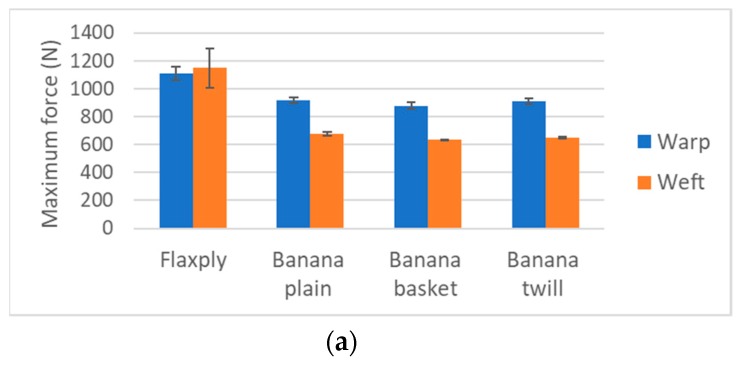

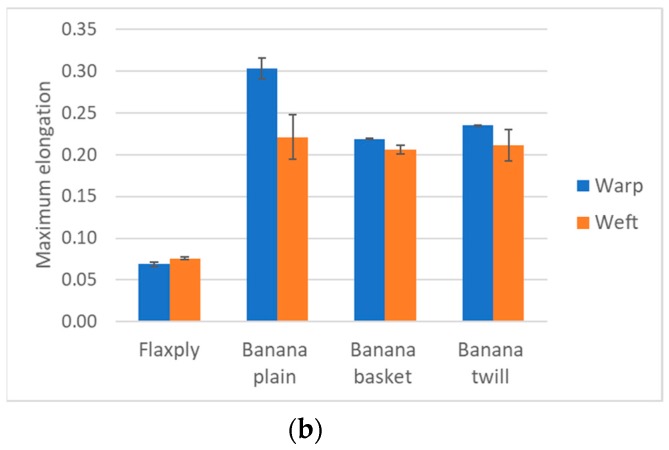
Maximum force and elongation of fabrics of banana fibre: (**a**)Maximum force; (**b**) Maximum elongation.

Different technical textiles were produced: plain, twill 3 × 1 and basket (Figure 1). In-tended weight was near 300 g/m^2^ and the textiles were produced in conventional lab-scale weaving devices (Texber, Avià, Spain) [20]. In order to compare the behaviour of the new textile, a commercial textile based on flax was used: Flaxply, 200 g/m^2^ (Lineo, Eco-Technilin, Valliquerville, France) with 2 × 2 twill structure. Five samples (200 mm × 50 mm) of each configuration were tested at the same equipment and storage conditions for 24 h mentioned before, with speed of testing 100 mm/min. Additionally, samples following the direction of warp and weft were tested as shown in Figure 2 [20].

### 2.2. Composite Material

The natural fibre composite with the new technical textile was processed by compression moulding. The polymeric matrix was polypropylene from Total Petrochemicals (PPH 9069).

Square 190 mm × 190 mm plates were produced by using different layers of textile and PP in a Collin Press P 200 PM hot-plate compression moulding machine (Dr. Collin GmbH, Ebersberg, Germany).

**Figure 3:**
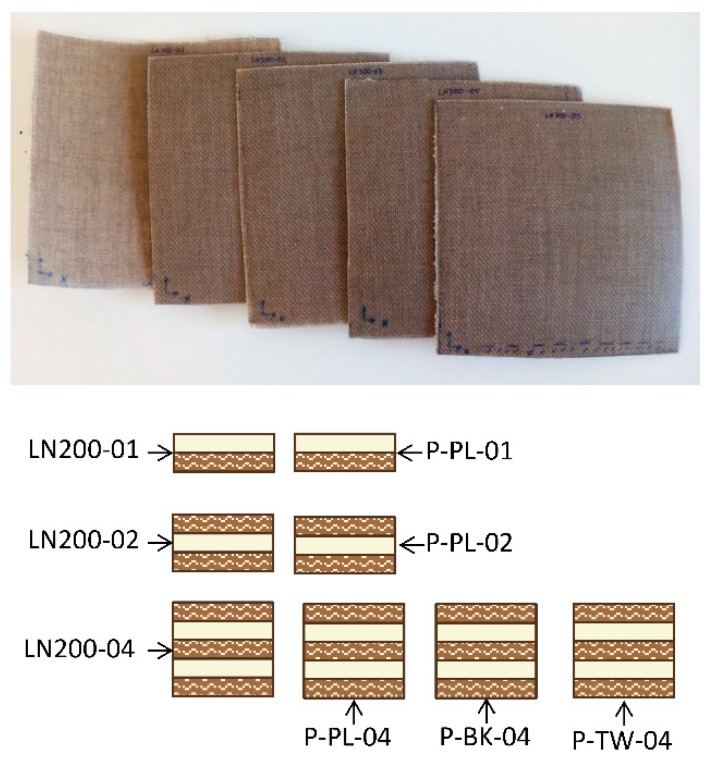
Configurations of composite (LN: flax; P: banana textile; PL: plain; BK: basket; TW: twill).

Different configurations of composite were done, modifying the number of sheets of textile and PP. For the purpose of the present paper the configurations are detailed in Figure 3, where the code LN is referred to flax textile (Lineo), P to banana textile, and PL, BK, and TW referred to plain textile, basket, and twill, respectively. The number of textiles per each configuration is 1, 2, or 3 and the number of PP sheets is 1, 1, and 2, respectively (1 mm thickness).

The parameters of each cycle time are shown in Table 1, being the degasification time, in stage 3, 10 s.

**Figure 4:**
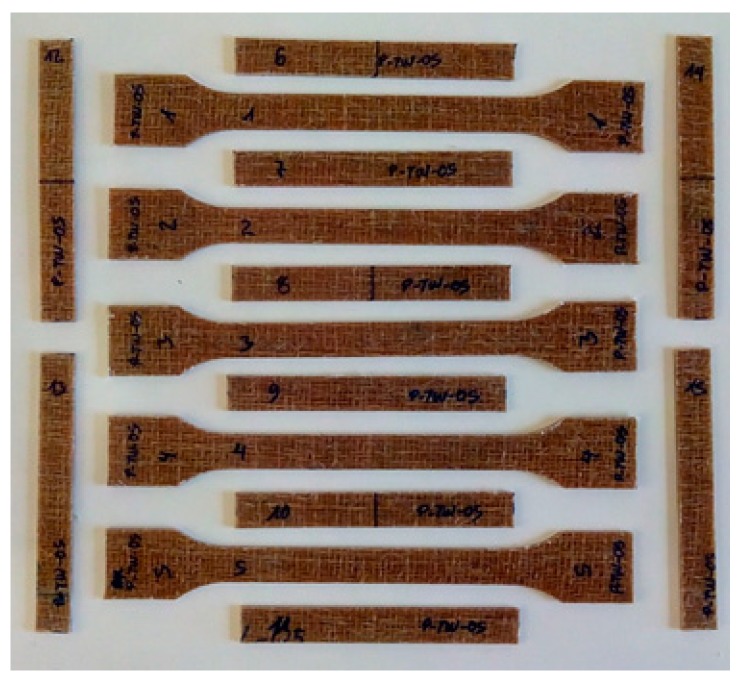
Tensile, flexural, and izod impact test.

From each plate 15 samples were machined in CNC milling machine (Mazak, Worcester, UK, Figure 4), keeping the direction of the warp for those under tensile test. The dimensions of the samples were according to the standards. The flexural and impact samples were 80 mm length and 10 mm width (the thickness depends on the manufacturing process and layers used). The tensile samples had the dimensions of 1A type according to the ISO 527-2 [21] (80 mm length and 10mm width in the testing zone, while the thickness depends on the number of layers and manufacturing process). The samples were tested following the standards ISO 527-2 [21] (tensile), ISO 178 [22] (flexural) and ISO 180 [23] (izod impact). Tensile tests were carried out in a machine with dynamometer YZC-516 (Electronics Wholesale Ltd, Guandong, China, 4000 N, 0.8 N resolution), with testing speed of 10 mm/min. For flexural, with dynamometer PCE-FB 50 (PCE, Meschede, Germany, 50 N, 0.01 N resolution), 50 mm/min. The impact test was done in LY-XJJD 50 Izod Impact Tester (Lyi Environmental Technology, Dongguan, China), with impact speed 3.5 m/s.

### 2.3. Methodology to Evaluate the Efficiency of the Developed Composite

One important aspect to study when a new composite is developed is the efficiency. The efficiency factor [7,24] shows the capacity of the textile or fibre to be integrated into the matrix, providing a composite material as a combination of the mechanical properties of both materials. A poor integration is consequence of several factors such as bad adherence due to small contact area matrix-fibre (for example clusters of fibres), bad physic and chemical bonding (some treatments should be required) or high level of voids (matrix cannot fill all the interstitial space of the textile). The determination of the efficiency factor gives us a good indication of the quality of the composite. In this study, the used parameters to calculate the efficiency factor are as follows:

(1) $$v_{f}=\frac{m_{f}}{d_{f}\times V_{c}}$$

(2) $$v_{r}=\frac{m_{c}-m_{f}}{d_{r}\times V_{c}}$$

(3) $$v_{v}=1-v_{r}-v_{f}$$

where *v*_f_: volume fraction of fibre; *v*_r_: volume fraction of matrix; *v*_v_: volume fraction of voids; *m*_f_: mass of fibre; *m*_c_: mass of composite; *d*_f_: density of fibre; *d*_r_: density of matrix; and *V*_c_: volume of composite. 

To determine the efficiency factor, the classic linear model was used [7,24], whereby for unidirectional fibre under tensile load the expression is:

(4) $$E_{c}=\left(E_{r}\times v_{r}\right)+\left(E_{f}\times v_{f}\right)$$

(5) $$\sigma_{c}=\left(\sigma_{r}\times v_{r}\right)+\left(\sigma_{f}\times v_{f}\right)$$

where *E*_c_: Young’s modulus of composite; *E*_r_: Young’s modulus of matrix; *E*_f_: Young’s modulus of the fabric; σ_c_: maximum stress of composite; σ_r_: maximum stress of matrix; σ_f_: maximum stress of the fabric.

To calculate the efficiency factor either in young modulus or maximum stress, the following equations were used:

(6) $$\varepsilon_{E}=\frac{E_{c}\;-\;E_{r}\;\times \;v_{r}}{v_{f}\;\times \;E_{f}\;\times \;O}$$

(7) $$\varepsilon_{\sigma }=\frac{\sigma_{c}-\sigma_{r}\times v_{r}}{v_{f}\times \sigma_{f}\times O}$$

The factor *O* is the orientation factor, which value is 1 for unidirectional fibre as the main direction. In this work, since the mechanical properties of the fibre have been obtained directly from the test of bidirectional textile (not from the individual yarn), the orientation factor has been fixed to 1. The reason for this is because the test of the textile already includes the effect of the transversal fibre.

Another criterion is to estimate the value of effective young modulus and maximum stress contributed by the fibre to the composite:

(8) $$E_{E}=\frac{E_{c}-V_{r}\times E_{r}}{V_{f}}$$

(9) $$\sigma_{E}=\frac{\sigma_{c}-V_{r}\times \sigma_{r}}{V_{f}}$$

## 3. Results and Discussion

### 3.1. Experimental Results

**Figure 5:**
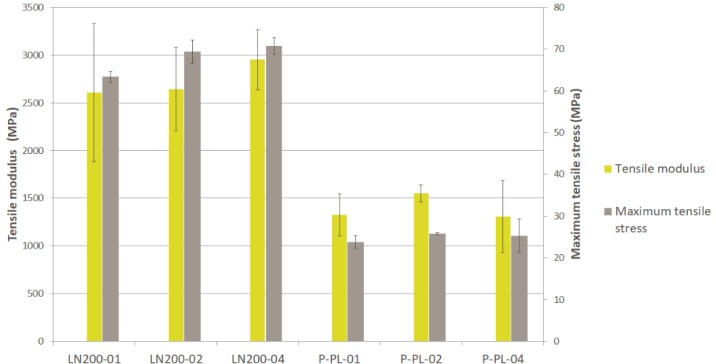
Tensile modulus and maximum stress of different composites.

**Figure 6:**
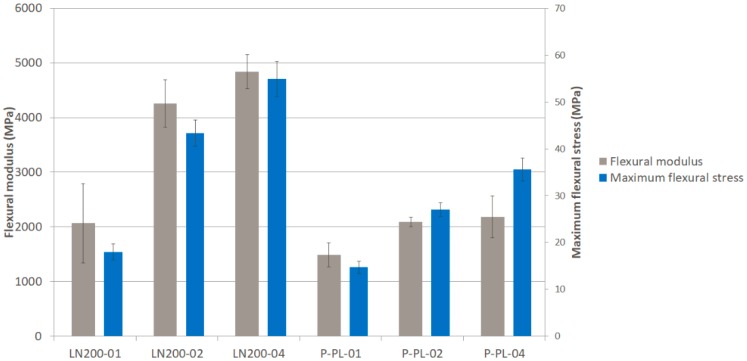
Flexural modulus and maximum flexural strength of different composites.

**Figure 7:**
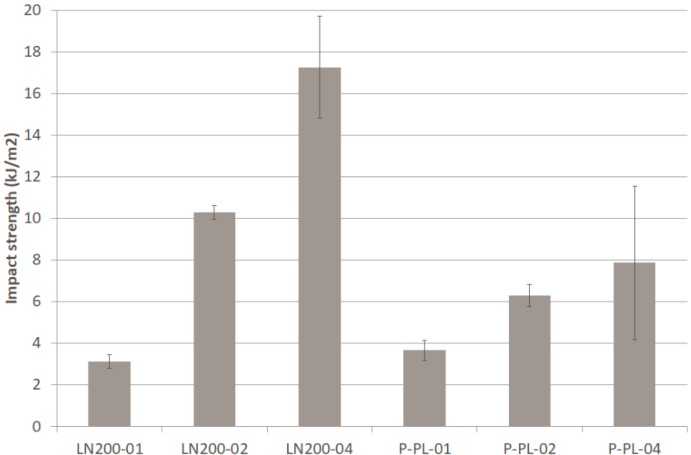
Impact strength of different composites.

Several configurations of the new developed textile were compared to the same performance made with commercial flax textile. Figure 5 (tensile modulus and maximum stress), Figure 6 (flexural modulus and maximum stress) and Figure 7 (impact strength) show the results for the different composites presented in Section 2. The typical dispersion of the results, in natural fibre composites, is observed as expected. Textile of flax is clearly better than the one of banana fibre in most of the tested parameters. The presence of wool reduces significantly the mechanical properties of the banana textile (50% of fibre is wool). The reason of adding this amount of wool to the yarn was the difficulty to process pure banana fibre with the available equipment; further trials will be done in the future and it is expected to achieve almost 100% of pure banana fibre, thus enhancing the mechanical properties.

In terms of strain at break for tensile, the configurations with flax textile kept near 4% and those with banana textile around 3%.

In order to verify whether or not the number of layers of the composite as well as the configuration of the fabric (plain, basket, twill) affect the tensile modulus and maximum strength, an ANOVA and Kruskal–Wallis tests were applied. ANOVA analysis determines the significance of one factor (e.g., configuration) on the average of a response variable (e.g., tensile modulus). The Kruskal–Wallis test is the equivalent non-parametric test, which means that the significance is evaluated on the median of a response variable. If the p-value is lower than 0.05, the corresponding factor is significant on the variation of the response variable (with 95% confidence interval). In Table 2 is seen the summary of this one way ANOVA and Kruskal–Wallis analysis, concluding that, in general, neither the number of layers nor the configuration of the fabric has significant influence on the tensile modulus and maximum stress in the composite with banana fibre. The only significant difference is observed in the maximum stress when the number of layers is modified, in particular between 01 and 02 or 01 and 03 as confirmed in the multiple range test (02 and 03 are homogenous groups so no difference between them).

**Figure 8:**
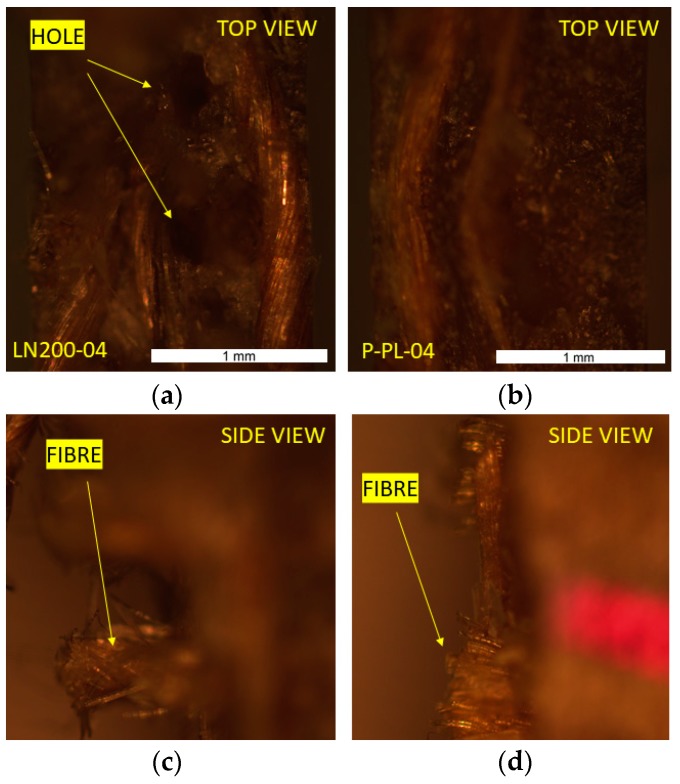
Section of tensile sample break. (**a**) Top view LN200-04; (**b**) Top view P-PL-04; (**c**) Side view LN200-04; (**d**) Side view P-PL-04.

The section fracture of the tensile sample is observed in Figure 8, where in the case of LN200-04 the process of pull-out of the fibre from the matrix seems to be more significant than the case of P-PL-04 (deeper holes and longer fibres pulled out).

To determine the efficiency factor of the composite, according to the methodology of Section 2.3, the considered parameters regarding the yarn and textile are shown in Table 3. Note that to calculate the volume fraction of fibre the diameter of banana fibre is the equivalent diameter formed by the filament of banana and the twisted filament of polypropylene (0.36 mm). The second consideration to take into account is that since the mechanical properties of the fibre are based on the test of the textile, the orientation factor is 1 as mentioned in Section 3.2.

The values of efficiency factor of the composite in terms of tensile modulus (ε*_E_*) and maximum stress (ε_σ_) are calculated in Table 4, as well as the effective tensile modulus (*E_E_*) and effective maximum stress (σ*_E_*), transferred from the fibre to the composite (Table 4). The density of fibre in textile is 1.7 g/cm^3^ for the flax and 1.5 g/cm^3^ for banana.

**Figure 9:**
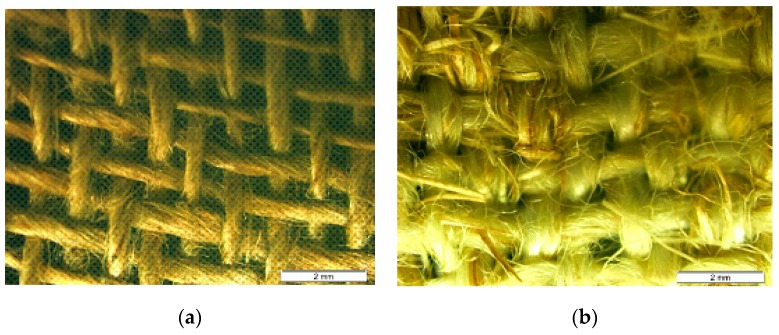
Spacing of warp/weft in flax textile (**a**) and banana textile (**b**).

The results of efficiency are relatively high in terms of efficiency of *E* modulus (ε*_E_*) having an average value of 0.86 for flax composite and 0.52 for banana composite (40% worse). Nevertheless, the most significant difference is in terms of efficiency of maximum stress (ε_σ_), with an average value of 0.52 for flax and 0.05 for banana (90% worse). The maximum stress is a consequence of the quality of adherence fibre-matrix, which depends on many factors such as the physico-chemical integration fibre-matrix, effective contact surface and level of voids. The analysis of the efficiency factor gives us a relevant information in terms of volume fraction of voids v_v_ (Table 4), where the volume fraction in the banana composite is the double (0.16) of the one of flax composite (0.08). This high level of voids in the banana composite means that the polymeric matrix could not fill all the holes of the textile during the compression moulding. The reason for this is observed in Figure 9, comparing the spacing warp/weft of the flax textile with the spacing of the banana textile. The holes of the banana textile are tighter because the yarn of banana is flatter (more elliptic) into the textile and less homogeneous. The second reason is the likely functionalization of the flax textile, producing a better integration matrix-fibre [25]. One issue that requires further analysis is the effect of the multifilament of PP twisted around the banana yarn. This PP, with different characteristics to the PP of matrix, could produce a negative effect in the compatibility fibre-matrix. Taking into account the good behaviour of banana fibre [4] in other composites, a second issue to study is the replacement of wool by other natural fibre. In this case, it is clear that the behaviour of the developed banana textile in terms of maximum stress is very poor because of the presence of wool.

### 3.2. Numerical Prediction of Mechanical Behaviour of Developed Composite

One relevant challenge when a new composite material is developed, is to provide the option of design and analysis with this new material. Since composites with technical textile are multi-material and non-isotropic, a methodology to simulate and predict the behaviour under specific loads is basic for real implementation in the industry. Finite element analysis (FEA) is very well known for analysis of solid bodies under mechanical or thermal loads and most of the commercial software allow the introduction of multi-material with different mechanical properties. Nevertheless, to apply this conventional method to this kind of composites could take too much computing time due to the need of meshing the full net of small filaments. This is the reason to use a commercial software such as Multiscale Designer^TM^ [17], allowing one single unit cell simulation of the selected fabric with a matrix, thus generating a composite at the micro-mechanic level. This unit of composite lamina has orthotropic properties and it is defined by the basic geometry of the technical textile. Once this single cell is characterized, the following step is to use it as a basic unit of the full body part, suitable to be analysed in conventional FEA with less CPU time and high level of reliability.

Multiscale Designer^TM^ creates parametric unit cells for different types of fibre composites from the user experimental data of the individual materials (e.g., matrix and reinforcement).

The simulated composite materials are the following:

- Plane fabric with banana threads and polypropylene matrix (P-PL-04)
- Twill fabric with banana threads and polypropylene matrix (P-TW-04)
- Basket fabric with banana threads and polypropylene matrix (P-BK-04)

**Figure 10:**
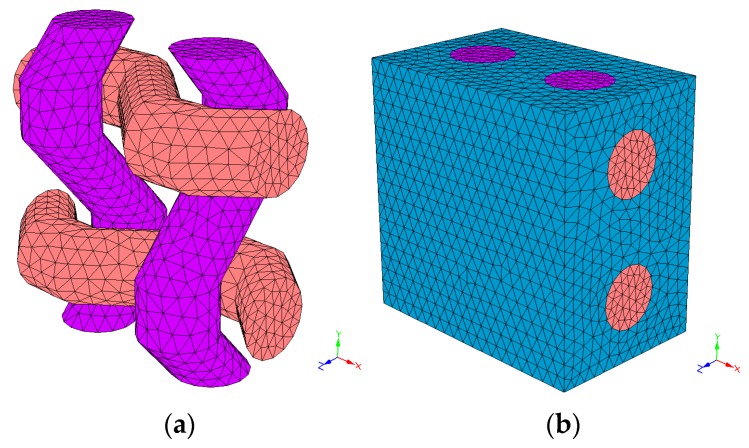
Plane fabric without PP (**a**) and with PP (**b**).

**Figure 11:**
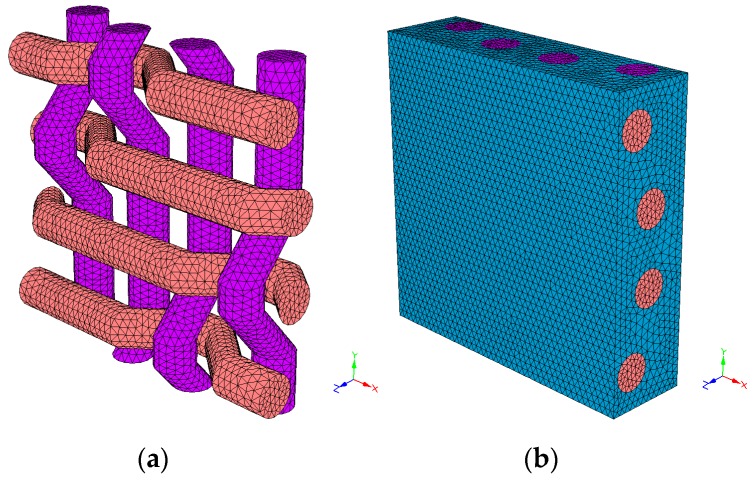
Twill fabric without PP (**a**) and with PP (**b**).

**Figure 12:**
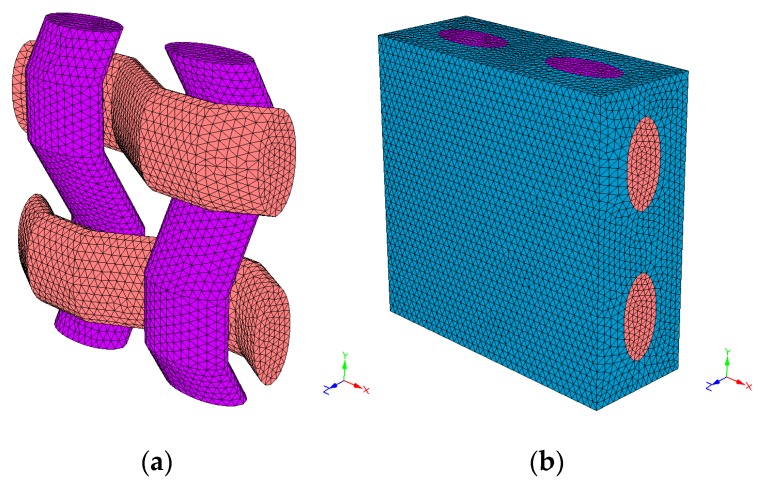
Basket fabric without PP (**a**) and with PP (**b**).

To simulate the lamina of composite material, it is necessary to know the geometry and properties of the fabric and the matrix. The necessary data at a geometrical level has been: two radiuses to define the yarn section, the distance in-between the weft yarns (X-axis) and in between the warp yarns (Y-axis), the fabric’s volume percentage and the textile structure. These data have been used to obtain the composite material models: P-PL (Figure 10), P-TW (Figure 11) and P-BK (Figure 12). Regarding the material properties, the following has been used: the Young’s modulus and the Poisson’s ratio of the matrix, the weft and the warp.

Due to the impossibility to generate the basket structure using the selected software (it did not allow the desired geometry), the most similar structure provided has been used (Figure 10). A plane with elliptic yarns to emulate the presence of two yarns has been used to substitute the basket.

The thread section within the composite material is elliptic because it is submitted to tractions and compressions generated by the textile structure and the matrix. The spaces have been measured on the fabric and the volume percentages have been calculated from the available data. In Table 5, the geometrical properties used to simulate the unit cell of each fabric are listed. No voids have been considered in the simulation.

For the matrix, an elastic modulus of 1600 MPa and a Poisson ratio of 0.42 have been used [26]. On the other hand, the Young’s modulus and Poisson’s ratio used for the yarns have been 669 MPa and 0.3 respectively. The yarns’ Poisson’s ratios have been obtained from other natural fibre studies [27]. The dimensions of the structural unit were computed by the software through the inputs depicted in Table 5. The software accomplishes the discretization by using four-node linear tetrahedral elements. Linear elastic properties were defined both for the matrix and yarns. With this data, the software computed the reduced order model, thus obtaining the linear orthotropic properties for the unit cell.

Through the simulation, the properties of the unit cell have been obtained and they have been extrapolated to a composite material lamina. The resulting lamina properties for each fabric type are listed in Table 6.

**Figure 13:**
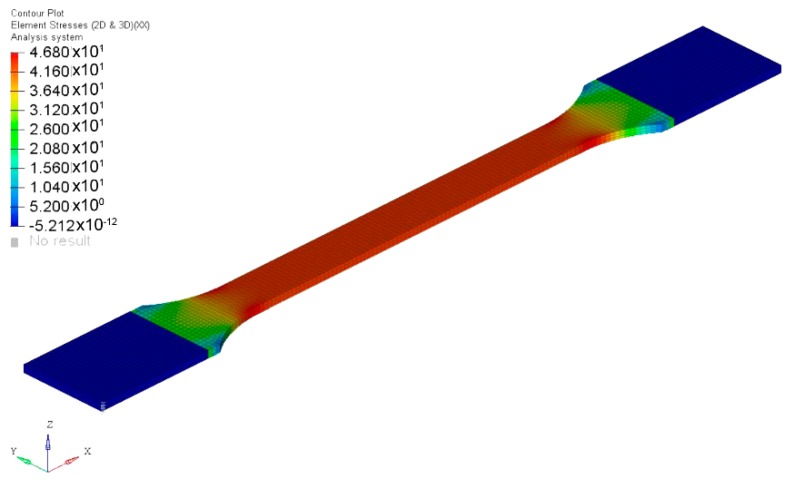
Element stresses (MPa): Direction X.

**Figure 14:**
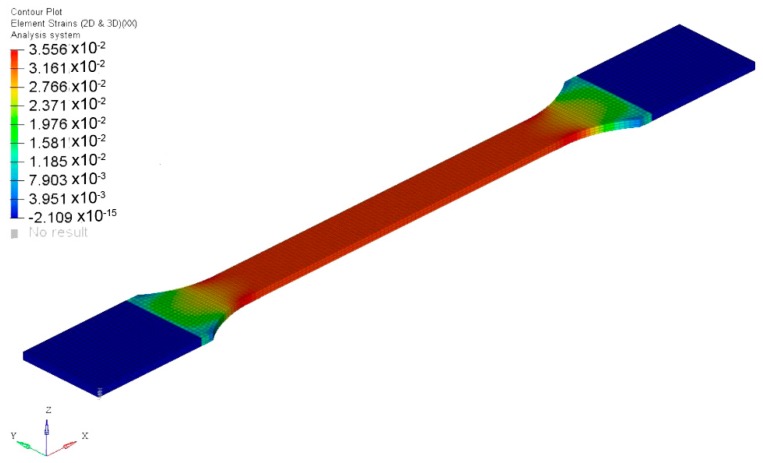
Element strains: Direction X.

Tensile samples according to the standard ISO 527-2:2012 [21] were modelled and the unit cell properties previously obtained were introduced for each material definition. The boundary conditions of the tensile tests were applied (one end of the sample without displacements, corresponding to the fixed clamp, and the other end with a longitudinal displacement, corresponding to the mobile clamp). Once the simulation was carried out, the stresses and strains in the longitudinal direction (*X* axis) were obtained in the middle zone of the sample (Figure 13 and Figure 14). Although the values were practically constant, the average value of stress and strain was calculated to compare the simulations with the experimental results.

The modulus in the direction X (E11), corresponding to the main direction of the tensile samples, was compared with the experimental results for each fabric in tensile testing. The error of the characterization of the individual cell is observed in Table 7.

The average error for the three different fabrics is about 9% in the elastic region of the material, which is acceptable taking into account the high typical deviation when composites with natural fibres are tested. Another conclusion is the confirmation of the results of ANOVA analysis mentioned in the previous experimental section, resulting that the values for tensile E11 are quite similar for the three different fabrics (variation coefficient of 0.4%). The characterization of the unit cell of each material can be used as an input for the following step in the design process (FEA of the 3D geometry of a real part).

## 4. Conclusions

This work presents the mechanical characterization of a new developed technical textile, with banana fibre, comparing experimental results with numerical simulation based on the definition of the unit cell at micromechanical level. Different configurations of the new composite were processed by compression moulding and compared to a commercial composite with flax fibre. Experimental results showed that no significant differences were observed between the mechanical properties of the different configurations (number of layers and fabric structure: plane, basket, or twill). The mechanical properties of the composite with flax were clearly higher than the new banana fibre composite. The main reason of this loss is the presence of wool (50%), as it is needed need to produce the continuous yarn, thus reducing the mechanical properties of the yarn. To explain the behaviour of each composite, a study of efficiency was done, determining that the efficiency of *E* modulus (ε_E_) for banana composite was 40% worse than the flax composite. On the other hand, the efficiency of maximum stress (ε_σ_) of banana is 90% worse than flax. Among other explanations for this poor result in terms of adherence fibre-matrix (such as functionalization of fibre, mix with wool, etc.), there is a clear cause that is the higher amount of voids (twice) in the banana composite, showing that the polymer did not reach many internal areas of the banana fabric. Nevertheless, further trials of this new banana yarn should be done, reducing or even removing all the wool to produce the yarn, thus improving the mechanical properties of the textile because banana fibre is well recognized as fibre with high mechanical performance.

In the second part of the research, a methodology to predict the behaviour of this new composite when applied to a real part is presented. In order to reduce the computing time and to simplify the process, a tool named Multiscale Designer was implemented as the first stage of the proposed methodology. In this first stage, the orthotopic properties of a single unit cell were calculated, starting from the properties of the matrix and banana yarn and designing the structure of each fabric (plain, basket, twill). Results of this numerical simulation had an acceptable error compared with the experimental results of the three composites, with a global average error of 9% in the elastic region. Additionally, the numerical simulation confirmed the conclusion of ANOVA analysis carried out with the experimental tests: there are not significant differences between the three structures of fabric in terms of mechanical properties.

## Figures and Tables

**Table 1:** Cycle of compression moulding.

Code	Stage	1	2	3	4	5
All	Upper and lower temperature of heater plates ( °C)	50	165	190	50	50
All	Pressure (Bar)	0	0	35	35	0
XX-01	Cycle time (s)	10	300	180	50	50
XX-02	Cycle time (s)	10	300	240	600	10
XX-04	Cycle time (s)	10	300	300	600	10

**Table 2:** One way ANOVA analysis and Kruskal–Wallis test.

Mechanical Properties	One-Way ANOVA *p* Value	Kruskal–Wallis Test *p* Value
Tensile modulus by layers	0.0455	0.0774
Tensile modulus by configuration	0.3922	0.3180
Maximum stress by layers	0.0018	0.0008
Maximum stress by configuration	0.0674	0.1222

**Table 3:** Basic parameters of textile considered for efficiency factor.

Fabric	Diameter Filament (Pre-Stressed) (mm)	Density Filament (g/cm^3^)	Tex	Tensile Modulus (MPa)	Maximum Stress (MPa)
**Textile LN200**	0.27	1.7	94	8825	373
**Textile P-PL**	0.25	1.5	157	3600	420

**Table 4:** Summary of efficiency factor of young modulus and maximum stress of composite. We re-edit the Table, please confirm.

Composite	*E*_c_ (MPa)	σ_c_ (MPa)	% Mass Fibre	% Mass Matrix	*d*_c_ (g/cm^3^)	*m*_c_ (g)	*m*_f_ (g)	*V*_c_ (mm^3^)	*v*_f_	*v*_r_	*v*_v_	ε_σ_	ε*_E_*	*E_E_* (MPa)	σ_E_ (MPa)
**LN200-01**	2607	63	33	67	0.95	0.50	0.17	526	0.19	0.72	0.09	0.57	0.88	7757	211
**LN200-02**	2642	69	35	65	0.99	0.92	0.32	927	0.21	0.72	0.07	0.58	0.81	7108	216
**LN200-04**	2951	71	40	60	0.99	1.25	0.50	1263	0.23	0.68	0.09	0.55	0.90	7978	207
**P-PL-01**	1323	24	41	59	0.92	0.56	0.23	608	0.24	0.62	0.14	0.03	0.38	1367	14
**P-PL-02**	1550	26	52	48	0.94	0.88	0.45	928	0.32	0.52	0.17	0.06	0.63	2284	27
**P-PL-04**	1475	25	52	48	0.95	1.31	0.68	1379	0.32	0.52	0.16	0.06	0.56	2017	23

**Table 5:** Geometrical properties of each fabric.

Dimensions	P-PL	P-TW	P-BK
Major Radius (mm)	0.3	0.3	0.6
Minor Radius (mm)	0.17	0.17	0.17
Spacing Weft (mm)	1.26	1.26	2.53
Spacing Warp (mm)	1.44	1.45	2.89
Volume %	30.49	30.36	30.38

**Table 6:** Lamina orthotropic properties for each unit cell.

Parameters	P-PL	P-TW	P-BK
E11 (MPa)	1286.7	1283.9	1294.2
E22 (MPa)	1288.8	1286.4	1295.6
E33 (MPa)	1245.4	1238.1	1227.4
V12	0.384	0.383	0.388
V23	0.384	0.384	0.381
V31	0.371	0.369	0.361
G12 (MPa)	453.2	451.6	454.7
G23 (MPa)	449.1	447.	438.9
G31 (MPa)	449.2	447.1	438.8

**Table 7:** Comparison between experimental tensile test and E11 of the unit cell.

Composite	Experimental Tensile Modulus (MPa)	Typical Deviation (MPa)	Unit Cell E11 (MPa)	Error (%)
P-PL-04	1307.2	379	1286.7	−1.6
P-TW-04	1051.1	63.3	1283.9	+22.1
P-BK-04	1238.0	172.5	1294.2	+4.5

## References

[1] Fifield L.S., Simmons K.L. Compression molded, bio-fiber reinforced, high performance thermoset composites for structural and semi-structural applications. Proceedings of the 10th Annual Automotive Composites Conference & Exhibition.

[2] Pothan L.A., Thomas S., Neelakantan N.R. (1997). Short banana fiber reinforced polyester composites: mechanical, failure and aging characteristics. J. Reinf. Plast. Compos..

[3] Monzón M.D., Suárez L.A., Pestana J.D., Ortega F., Benítez A.N., Ortega Z., Hernández P.M., Marrero M.D., Diaz N., Paz R., Casas A., Artal G. (2014). Procedure and machine for producing fiber from leaves. Patent.

[4] Ortega Z., Morón M., Monzón M.D., Badalló P., Paz R. (2016). Production of banana fiber yarns for technical textile reinforced composites. Materials.

[5] Kalia S., Kaith B.S., Kaur I. (2009). Pretreatments of natural fibers and their application as reinforcing material in polymer composites—A review. Polym. Eng. Sci..

[6] Cherif Z.E., Poilâne C., Falher T., Vivet A., Ouail N., Doudou B.B., Chen J. (2013). Influence of textile treatment on mechanical and sorption properties of flax/epoxy composites. Polym. Compos..

[7] Mehmood S., Madsen B. (2012). Properties and performance of flax yarn/thermoplastic polyester composites. J. Reinf. Plast. Compos..

[8] Muralidhar B.A. (2013). Tensile and compressive properties of flax-plain weave preform reinforced epoxy composites. J. Reinf. Plast. Compos..

[9] Dhakal H., Zhang Z., Bennett N., Lopez-Arraiza A., Vallejo F. (2014). Effects of water immersion ageing on the mechanical properties of flax and jute fibre biocomposites evaluated by nanoindentation and flexural testing. J. Compos. Mater..

[10] Byun J.-H., Chou T.-W. (1989). Modelling and characterization of textile structural composites: A review. J. Strain Anal. Eng. Des..

[11] Tan P., Tong L., Steven G.P. (1997). Modelling for predicting the mechanical properties of textile composites—A review. Compos. Part A: Appl. Sci. Manuf..

[12] Ayranci C., Carey J. (2008). 2D braided composites: A review for stiffness critical applications. Compos. Struct..

[13] Le Page B.H., Guild F.J., Ogin S.L., Smith P.A. (2004). Finite element simulation of woven fabric composites. Compos. Part A: Appl. Sci. Manuf..

[14] Fish J. (2013). Practical Multiscaling.

[15] Verpoest I., Lomov S.V. (2005). Virtual textile composites software WiseTex: Integration with micro-mechanical, permeability and structural analysis. Compos. Sci. Technol..

[16] Lomov S.V., Verpoest I., Peeters T., Roose D., Zako M. (2003). Nesting in textile laminates: geometrical modelling of the laminate. Compos. Sci. Technol..

[17] Yuan Z., Crouch R., Wollschlager J., Fish J. (2017). Assessment of multiscale designer for fatigue life prediction of advanced composite aircraft structures, Assessment of multiscale designer for fatigue life prediction of advanced composite aircraft structures. J. Compos. Mater..

[18] UNE-EN ISO 2062-2010 (2010). Textiles - Yarns from Packages - Determination of Single-end Breaking Force and Elongation at Break Using Constant Rate of Extension.

[19] EN ISO 139 Textile - Standard Atmospheres for Conditioning and Testing. https://www.din.de/en/wdc-beuth:din21:140644768.

[20] Ortega Z., Monzón M., Paz R., Suárez L., Morón M., McCourt M., Campana G., Howlett R.J., Setchi R., Cimatti B. (2017). Banana Fiber Processing for the Production of Technical Textiles to Reinforce Polymeric Matrices. Proceedings of the Sustainable Design and Manufacturing 2017.

[21] ISO 527-2 Plastics - Determination of Tensile Properties - Part 2: Test Conditions for Moulding and Extrusion Plastics. https://www.iso.org/standard/56046.html.

[22] ISO 178 Plastics—Determination of Flexural Properties. https://www.iso.org/obp/ui/#iso:std:iso:178:ed-5:v1:en.

[23] ISO 180 Plastics—Determination of Izod Impact Strength. https://www.instron.us/en-us/testing-solutions/by-material/plastics/impact/iso-180.

[24] Wang W., Ciselli P., Kuznetsov E., Peijs T., Barber A.H. (2008). Effective reinforcement in carbon nanotube–polymer composites. Philos. Trans. R. Soc. A Math. Phys. Eng. Sci..

[25] Raemdonck J.V. (2011). Method for preparing thermosetting or thermoplastic polymer or elastomer composites that are reinforced with natural fibers, and their multiple applications as construction material. U.S. Patent.

[26] Typical Engineering Properties of Polypropylene. https://studylib.net/doc/18216879/typical-engineering-properties-of-polypropylene.

[27] Ramakrishnan S., Krishnamurthy K., Prasath M.M., Kumar R.S., Dharmaraj M., Gowthaman K., Kumar P.S., Rajasekar R. (2015). Theoretical prediction on the mechanical behavior of natural fiber reinforced vinyl ester composites. Appl. Sci. Adv. Mater. Int..

